# Embedding knowledge on ontology into the corpus by topic to improve the performance of deep learning methods in sentiment analysis

**DOI:** 10.1038/s41598-021-03011-6

**Published:** 2021-12-07

**Authors:** Duy Ngoc Nguyen, Tuoi Thi Phan, Phuc Do

**Affiliations:** 1 Faculty of Computer Science and Engineering, Ho Chi Minh City University of Technology (HCMUT), Ho Chi Minh City, 72506 Vietnam; 2 Vietnam National University at Ho Chi Minh City, Ho Chi Minh City, Vietnam; 3 Faculty of Information Technology, Posts and Telecommunications Institute of Technology, Ho Chi Minh City, 71007 Vietnam; 4 Faculty of Information Systems, University of Information Technology, Vietnam National University Ho Chi Minh City, Ho Chi Minh City, 71308 Vietnam

**Keywords:** Computer science, Scientific data, Computational science

## Abstract

Sentiment classification, which uses deep learning algorithms, has achieved good results when tested with popular datasets. However, it will be challenging to build a corpus on new topics to train machine learning algorithms in sentiment classification with high confidence. This study proposes a method that processes embedding knowledge in the ontology of opinion datasets called knowledge processing and representation based on ontology (KPRO) to represent the significant features of the dataset into the word embedding layer of deep learning algorithms in sentiment classification. Unlike the methods that lexical encode or add information to the corpus, this method adds presentation of raw data based on the expert’s knowledge in the ontology. Once the data has a rich knowledge of the topic, the efficiency of the machine learning algorithms is significantly enhanced. Thus, this method is appliable to embed knowledge in datasets in other languages. The test results show that deep learning methods achieved considerably higher accuracy when trained with the KPRO method’s dataset than when trained with datasets not processed by this method. Therefore, this method is a novel approach to improve the accuracy of deep learning algorithms and increase the reliability of new datasets, thus making them ready for mining.

## Introduction

In the age of social media, opinions and sentiments are shared more frequently and widely than ever before. The number of Likes for opinions shared on social media tells us which topics are receiving the most attention, which in turn helps businesses and artists to understand what consumers think of their products. Therefore, the problem of sentiment classification for images or texts is of great interest^1–3^. Sentiments are often expressed very subtly, however, which means that building a system that can classify sentiment to a high level of reliability is a huge demand. This paper studies the performance improvement of machine learning algorithms in sentiment analysis users. Review comments on products in social networking platforms and forums are often not elaborate and accurate in terms of words and grammar. Therefore, training machine learning systems to understand these comments is a challenging task. To improve the performance of deep learning algorithms, studies have typically focused on enhancing the feature learning process of the algorithms. Feature computation is a significant problem that arises when determining the ability of machine learning methods. Numerous feature computation techniques, such as word2vec by Xin^4^, Global Vector (GloVe) by Pennington et al.^5^, and term frequency-inverse document frequency (TF-IDF) by Wu et al.^6^, have been introduced and are widely employed. Recently, Jacob et al.^7^ proposed the BERT model and used the data processing method, WordPiece by Wu et al.^8^; they achieved breakthrough results in natural language processing. Concerning sentiment classification, studies based on the BERT method, such as those by Lan et al.^9^ and Yang et al.^10^, have achieved high accuracies of over 95% on the SST-2 dataset by Socher et al.^11^. In 2014, Kim^12^ used a convolutional neural network (CNN) model for word embeddings created using word2vec and achieved an accuracy of 88.1%. In 2017, McCann et al.^13^ used GloVe to create word embeddings by adding a contextual vector built using a machine translation technique. They achieved an accuracy of 91.2%.

However, the aforementioned feature computation techniques were not as accurate on the IMDb dataset by Mass et al.^14^. In 2019, Rehman et al.^1^ combined the CNN and long-short term memory (LSTM) models and used word2vec to build a word embedding layer to perform sentiment classification. They achieved an accuracy of 91%. In 2020, Benlahbib and Nfaoui^15^ achieved an accuracy of 88.81%, which is lower than that achieved a year earlier by Rehman et al.^1^, although Benlahbib and Nfaoui^15^ added information to the corpus. Jang et al.^16^ used word embeddings built using word2vec and achieved an accuracy of 88.74% with a CNN, 89.40% with an LSTM, 71.29% with a multilayer perceptron, and 91.41% with a combined Bi-LSTM (Bi-directional long-short term memory) + CNN model. In 2017, non-neural network-based methods such as support vector machine (SVM) and Naïve Bayes Manek et al.^17^ achieved accuracies of 94.46% and 87.50%, respectively. Further, Manek et al.^17^ used the TF-IDF method to combine the Gini-index technique to construct feature vectors. However, in 2018, Kumar et al.^18^ used the TF-IDF method and achieved accuracies of 76.6% and 63.4% with the SVM and naïve Bayes, respectively. Gu et al.^19^ performed sentiment classification using the Amazon dataset with two polarities using a CNN and word embeddings created using word2vec and achieved an accuracy of 84.87%.

With less popular datasets such as TripAdvisor and BeerAdvocate or book review data, the accuracy of sentiment analysis has been shown to be limited. For instance, Yin et al.^20^ achieved an accuracy of 46.56% using a multi-channel LSTM hierarchical combination model to process each word in a sentence. Mukhlash et al.^21^ achieved an accuracy of 66.03% using a combined CNN and LSTM model. Bie and Yang^22^ proposed a system that combines multiple LSTM, GRU (Gated Recurrent Units), and CNN models to handle various tasks. When experimenting on the Laptop review datasets, the Restaurant and Twitter datasets did not achieve the same effect. The test results for the Restaurant dataset reached an F1 value of 65.20%, the Laptop dataset only achieved an F1 value of 55.08%, and the Twitter dataset achieved the lowest F1 value (just 47.89%). Zhai et al.^23^ encountered a similar situation when proposing a model combining many LSTM modules to conduct sentiment analysis for Course, Education, and Restaurant datasets. This model achieved an accuracy of 94.6% on the Education dataset. However, it scored less well on the other two datasets, achieving an accuracy of 81.4% on the Course dataset and 79.6% on the Restaurant dataset.

The abovementioned studies used word2vec, GloVe, TF-IDF, or WordPiece for performing data conversion of original datasets without making any changes to the data. Shah et al.^24^ added a dataset, which significantly increased the accuracy of their model. However, this method of adding information cannot be applied to all types of domain data. Duy et al.^25^ used deep learning algorithms, such as a CNN or an LSTM, which are considerably similar in terms of efficiency of learning datasets that have two topics in two different languages for sentiment classification. However, choosing the best calculation method and classification algorithm for a real sentiment analysis project from the methods considered earlier is a difficult task. The performance of the algorithm depends on the datasets. Each opinion dataset in each topic will have unique characteristics and will therefore only provides valid data for mining in a small selection of communities, even within the same topic. For example, the same car manufacturer's model may be evaluated by customers in different ways depending on the operating conditions and other conditions in each country. Thus, to appeal to customers in different markets, it is necessary to capture the unique characteristics of consumers in each market. Then, what to do is to build a new corpus. Determining the reliability of this new corpus and determining the appropriate computational method and machine learning algorithm is not an easy task. Because of this, it is formulating a method for building the corpus to train deep learning algorithms with high confidence is the motivation of this research.

Herein, we propose a data processing model that represents the knowledge of a data domain in the training dataset through the knowledge processing and representation based on ontology (KPRO) method to enhance deep learning methods for sentiment analysis. The first step in the KPRO method is the selection of terms representing the set of words or phrases that indicate the object aspect being evaluated. These terms receive words having contextual relationships, with facet-substituted words to enrich said context. Next, processing is performed to transform the long-distance contextual relations of this entity with sentiment words into close relations based on the generalization process of reviews. Through the above two steps, a set of terms with contextual relationships with other words is created in a rich and diverse corpus. The relationship between these terms and sentiment words is also highlighted. These features are evident in the word-embedding layer when built using tools such as word2vec. Components such as aspect words, sentiment words, and substituting terms for the aspect belong to an ontology, which is built on a corpus with expert knowledge of it. For example, car reviews form a topic. A car comprises numerous components. Car users are diverse; thus, their opinions regarding cars will also be very diverse from the viewpoint of words used. The complexity of the opinions regarding this topic will likely require a large amount of data for deep learning algorithms to learn its features if there exists no efficient data processing method. By appropriately embedding ontological knowledge into the training data, the data domain features can be made clearly visible, thereby enhancing the learning ability of deep learning algorithms even with a small corpus.

This study is based on the semantic and sentiment vocabulary hierarchical tree (SSVHT) ontology proposed by Duy et al.^26^. The SSVHT ontology represents the relationship between words or phrases indicating the standard aspect (used in car commercial websites); the user uses these words or phrases to review a particular aspect of the car and denote the user’s sentiment behind a specific review. The KPRO method searches for the component that expresses high-level concepts in the SSVHT ontology (aspect term class) to replace items representing low-level concepts in the SSVHT ontology (the same or approximately standard aspect class). Simultaneously, we indicate the relationship of this standard aspect with the sentiment terms in the sentiment term class.

To evaluate the effectiveness of the KPRO method, this study uses deep learning methods such as BERT, which is a robust and newly developed model; the LSTM and Bi_LSTM models for processes having long dependencies between word sequences; CNNs for extracting high-level features; and combined CNN-LSTM and CNN-Bi_LSTM models for exploiting the strengths of the CNN and LSTM or Bi_LSTM models. Machine learning methods that are not based on neural networks, such as the SVM and naïve Bayes, are also used to perform a complete assessment of the KPRO method.

The structure of the paper is as follows. The “Introduction” section outlines the background of the problems that motivated the research. The “Methodology” section presents the method we used to build and process embedding ontology into the corpus to enhance deep learning systems’ learning ability. The “Experiment” section introduces the test scenario and presents the result of sentiment classification after learning knowledge from the corpus, as processed by the KPRO method. The “Empirical evaluation” section evaluates the KPRO method, comparing its effectiveness with that of other methods. Finally, in the “Conclusion and future work” section, we conclude the paper and suggest avenues for future research.

## Methodology

Realizing the concept of the KPRO method requires a suitable corpus. The authors of this study possessed a detailed understanding of the field. Therefore, they were able to process data appropriately at different stages. This study used the Vietnamese corpus collected by the authors. This is a set of reviews of cars (car opinion in Vietnamese, COV).

### Introduction of the corpora used for the experiment

Automotive reviews were collected from online newspaper sites that record readers' opinions on websites on auto categories, auto-specific forums, and commodity business websites.

#### Building the set of car’s aspects

We chose the specifications often used by a manufacturer when introducing their product or the specifications the user is interested in while reviewing the car. Each aspect has an official name which is commonly used by manufacturers and familiar words that users use when expressing opinions on the Internet. For example, aspects are engine, interior, exterior, price, transmission, safety, etc.

#### Handle aspect analysis

Opinion reviews for more than one aspect were processed and split into numerous sentences corresponding to the number of aspects reviewed in the opinion. These sentences were evaluated in such a way that the original form was retained. The sentences belong to the opinion that is used to review a set of numerous aspects; each aspect in the sentence is divided into a new sentence that is technically appropriate for each aspect. These cases are illustrated in examples 2.1, 2.2, and 2.3.

Example 2.1: “Nội thất em này kém sang. Giá bán lại còn quá chát. Tổng thể không hấp dẫn*.*” *This furniture was less luxurious. The sale price was too high. Overall, it is not attractive.)*

The aspects reviewed are the interior, price, and overall aspects. The split aspect of the comments is as follows:

- “Nội thất em này kém sang*” (This car's interior is less luxurious)*
- “Giá bán lại còn quá chát” (*The price is too expensive)*
- “Tổng thể không hấp dẫn” (*Overall, it is not attractive*)

Example 2.2: “Nội thất ngon đấy tuy hơi kém sang còn giá thì quá chát*.*” (*The interior is fairly good but slightly less luxurious, and the price is too expensive*.)

Both the interior aspect and selling price aspect appear in the same sentence. Thus, this opinion is divided into the following sentences:

- “Nội thất ngon đấy tuy hơi kém sang” (*The interior is delectable but slightly less luxurious*).
- “giá thì quá chát” (*The price is too expensive*).

Example 2.3: “Mình thấy chiếc này nội thất hay ngoại thất gì cũng ngon.” (*In my opinion, the interior or exterior of the car is also delectable*.

Both interior and exterior aspects appear in the same sentence and share the adjective “delectable.” The aspects of this opinion are separated as follows:

- Sentence 1: “Mình thấy chiếc này ngoại thất cũng ngon” (*In my opinion, the exterior of this car is seemingly delectable*).
- Sentence 2: “Mình thấy chiếc này nội thất cũng ngon” (In my opinion, the interior of this car *is seemingly delectable*).

#### Assign sentiment labels to opinions

The opinions of car reviews are often diverse. An opinion may review only one aspect or may review multiple aspects. An opinion can be one sentence or more than one sentence. Labeling opinions from a review of multiple aspects is a complicated task. Users can positively alter one aspect and negatively alter another. Therefore, this paper sets out several criteria for the labeling of members reviews to reach a consensus and attain high consistency.

- Price is an important factor.
  This study collected reviews of popular cars. For users interested in this car, the price is an important factor affecting the customer's car purchase.
- Technical and technological factors are more important than esthetic ones.
  The safety and durability of a car depend on the technical and technological factors. These factors also affect other costs associated with the use of cars. Most users still consider technology and engineering to be more important than esthetic factors.
- Determination of sentiment labels based on opinion context.
  The car reviews are not only comments directed toward a car but can also involve comparisons between two cars, the form of which is very diverse. The corpus used in this study did not include explicit comparisons between two cars, but only comparisons between cars in articles where the main car in question is not clearly known; whereas, the one to which the main car is being compared to, is clearly known. The car in question may not appear in the comments.
  The corpus was labeled by two persons using Cohen's kappa coefficient by Jean^27^. K = 0.81. The coefficient K is determined by Eq. (1).
  1 $$ K = \frac{P(A) - P(E)}{{1 - P(E)}} $$
  where *P(A)*: the relative observed agreement among raters and *P(E)*: the hypothetical probability of chance agreement.
  After labeling the sentences, one aspect review will be based on the sentiment word or the semantics of that sentence.

### Construction of the ontology

**Figure 1:**
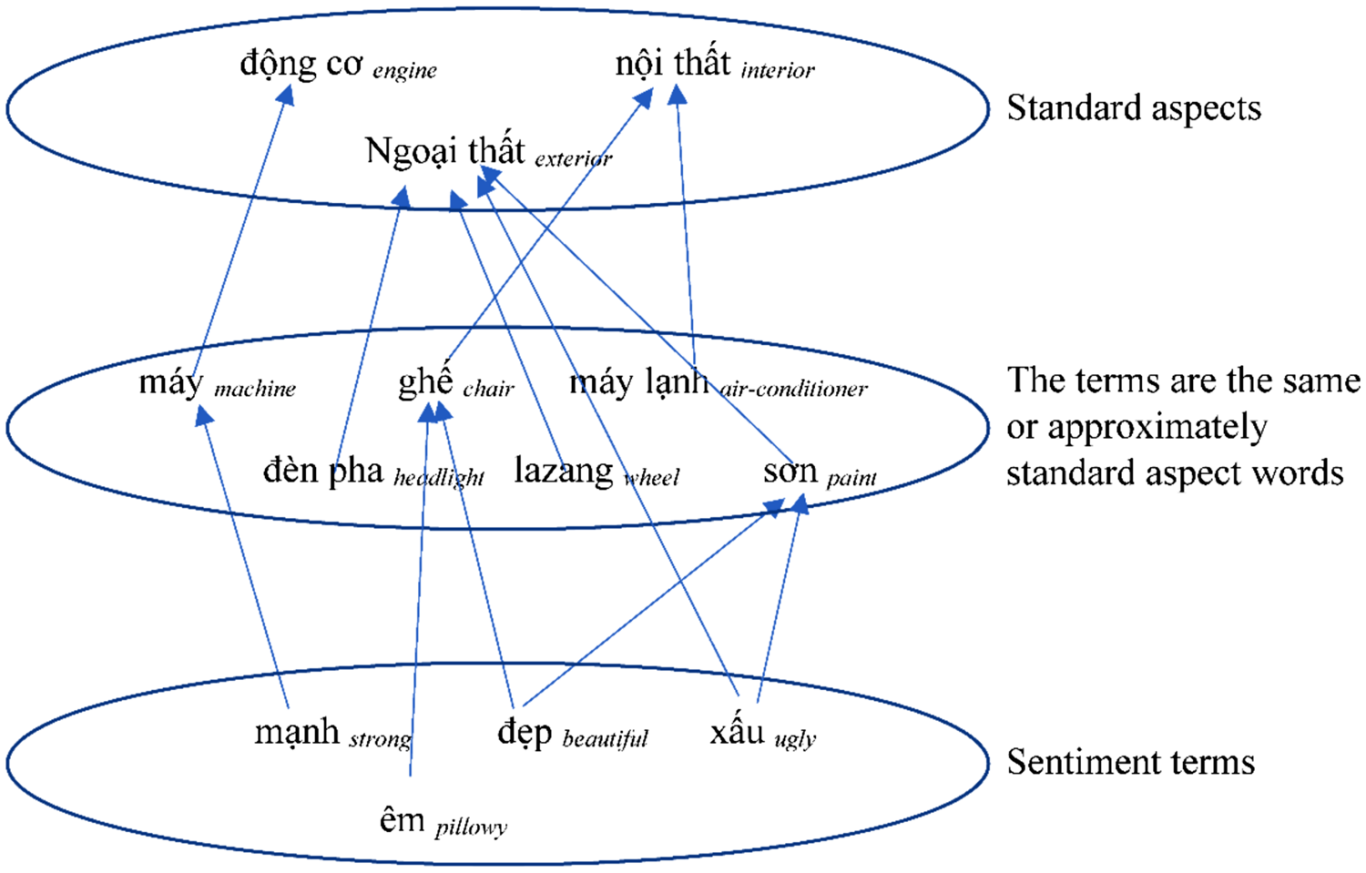
Semantic and sentimental ontology model of car reviews.

As mentioned in the above section, the ontology and construction in this study is based on the SSVHT ontology. The ontology has a class that defines the aspect terms used by the manufacturer, called standard aspects; a class that defines the aspect terms that the user uses, called the same or approximately standard aspect words; and a class that defines the user's sentiment terms to review aspects of the car, called sentiment words. Figure 1 shows the process of building the ontology model.

- Standard aspects
  Specifications are often classified by manufacturers and grouped into categories related to the technology applied to the product. We synthesized information of numerous car manufacturers and chose the general aspects introduced by most manufacturers, such as engine, performance, size, safety, exterior, and interior, as listed in the “Methodology” section.
- The same or approximately standard aspect words
  The user's comment may contain the car's specifications according to the manufacturer's terms or the user may use words with spoken language or slangs. For example, users can use the word “*máy *_*machine*_” for "*động cơ *_*engine*_,” “*la zang *_*wheel*_” for “*mâm *_*wheel*_” or “*lái *_*drive*_” for “*vận hành *_*transmission*_,” and “*lốp *_*tire*_” for “*vỏ *_*shell*_.” The words used as substitutes have the same or approximately standard aspect ((hereinafter referred to as "standard aspect equivalent")) terms by comparison of their meanings or refer to only a technical feature of the standard aspect terms. We identified standard aspect-equivalent terms in numerous ways:
  - Based on the part of speech: Labeling parts of speech by dependency grammar. Find a set of nouns/noun phrases (hereinafter referred to as terms) related to the technical characteristics of cars in the corpus's sentences.
  - Using the word2vec tool: The standard aspect equivalent word is enriched based on the vocabulary matrix built using word2vec.
  - Consultation from technical staff of the garage.
- Sentimental words
  The types of words used were adjectives, adverbs, and verbs modifying the terms that indicate the standard aspect word or the standard aspect equivalent word, which are sentiment terms contained in the opinion review. Sentimental words are single words that are often used when expressing positive or negative reviews with respect to cars; they are also called seed words. Example 2.4 illustrates some seed words used in car reviews.

Example 2. 4

- Engine: mạnh _strong_/yếu _weak_, bốc _impetuous_/í _sluggish_, and so on.
- Overall: hầm hố _robust_/ẻo lả _flabby_, hấp dẫn _attractive_/chán _forbidding_, and so on.

Depending on the perception, users can evaluate aspects at different levels based on the word sentiment combined with the degree elements. Thus, a phrase indicating the increasing or decreasing sentiment of seed words is formed. These complementary words are called derivative seed words, for example, too expensive and very powerful. These adverbs were divided into five groups, namely **intensifier**, **booster**, **diminisher**, **minimizer**, and **not** by Bang^28^. Sentiment terms may be enriched. The difference in the degrees of the "đắt (*expensive*)" adjective combined with modifiers is shown in Example 2.5.

Example 2.5: **cực kỳ** đắt (**extremely** expensive) > **thật** đắt (**too** expensive) > đẹp > **khá** đắt (**rather** expensive) > **cũng** đắt (**seemingly** expensive) > **không** đắt (**not** expensive).

This study uses word2vec to enrichment sentiment terms.

### Embedding ontology into the corpus

Details of the corpus before ontology embedding is shown in Table 1.

**Table 1:** Organization of the corpus.

Features of corpus	Quantity
Sample of cars	121
Opinions	2994
Sentences	9051
Sentiment labels	3 (positive, neutral, negative)
Sentences positive label	3487
Sentences neutral label	2820
Sentences negative label	2744
Standard aspect terms	10
Aspect terms	101
Number of words in the dataset	248,258
Sentiment term	3785

**Figure 2:**
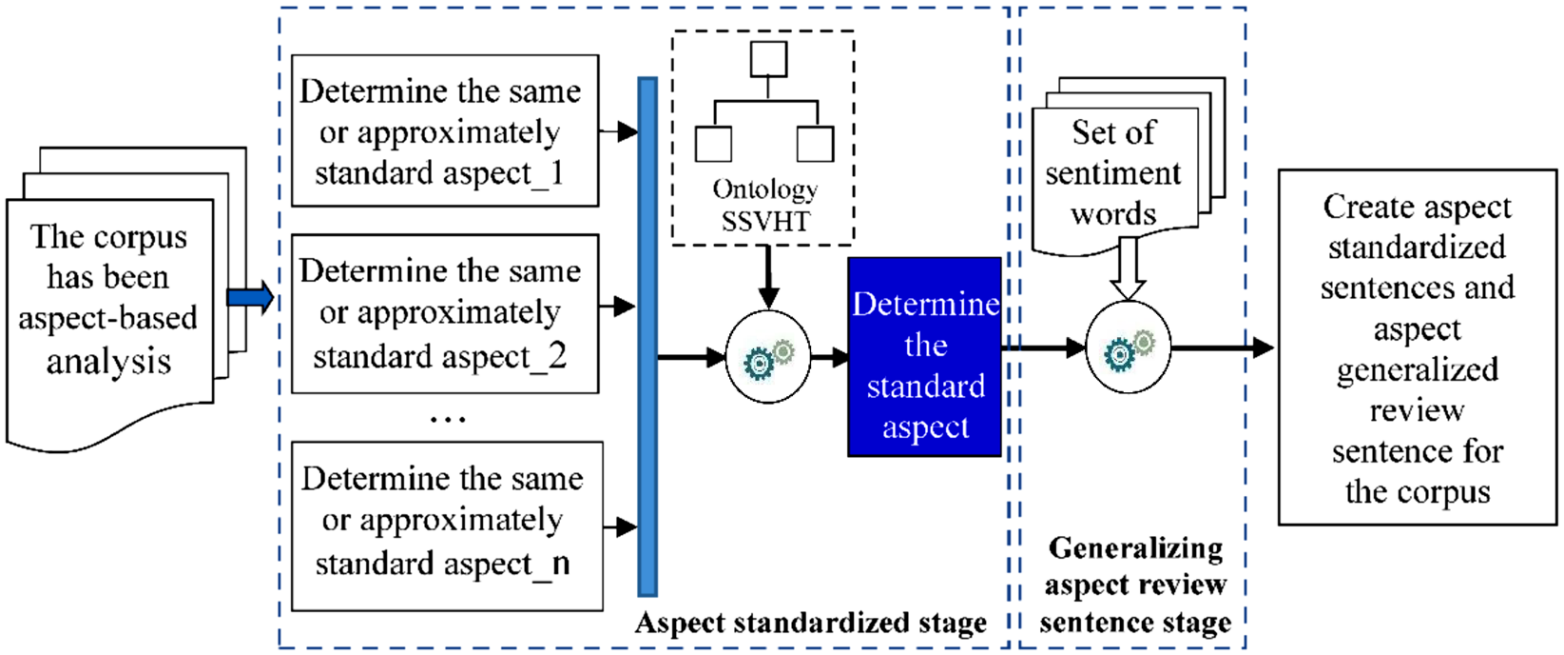
The model embeds knowledge in the corpus.

The data in Table 1 show that this is not a large corpus. The SSVHT ontology was embedded into the COV corpus to enrich the knowledge of this corpus. The model for embedding the ontology into the corpus is shown in Fig. 2.

The steps in this process are as follows:

Step 1: Aspect standardization.
  This step explores the ontology element that represents a higher-level aspect concept, replacing the current aspect element.
Step 2: Generalizing aspect review sentences.
  Users can express their reviews in different ways. However, it is possible to summarize the user's review as a compliment and criticize that aspect's details. This generalization process expresses the compliments and criticism of the standard aspects of the car, instead of the detailed aspects.
Step 3: Represent the aspect and sentiment relationships of the SSVHT ontology into the corpus.
  This is the process of adding knowledge to a corpus; the comments generated by Steps 1 and 2 are added to the corpus.

#### Aspect standardization

**Figure 3:**
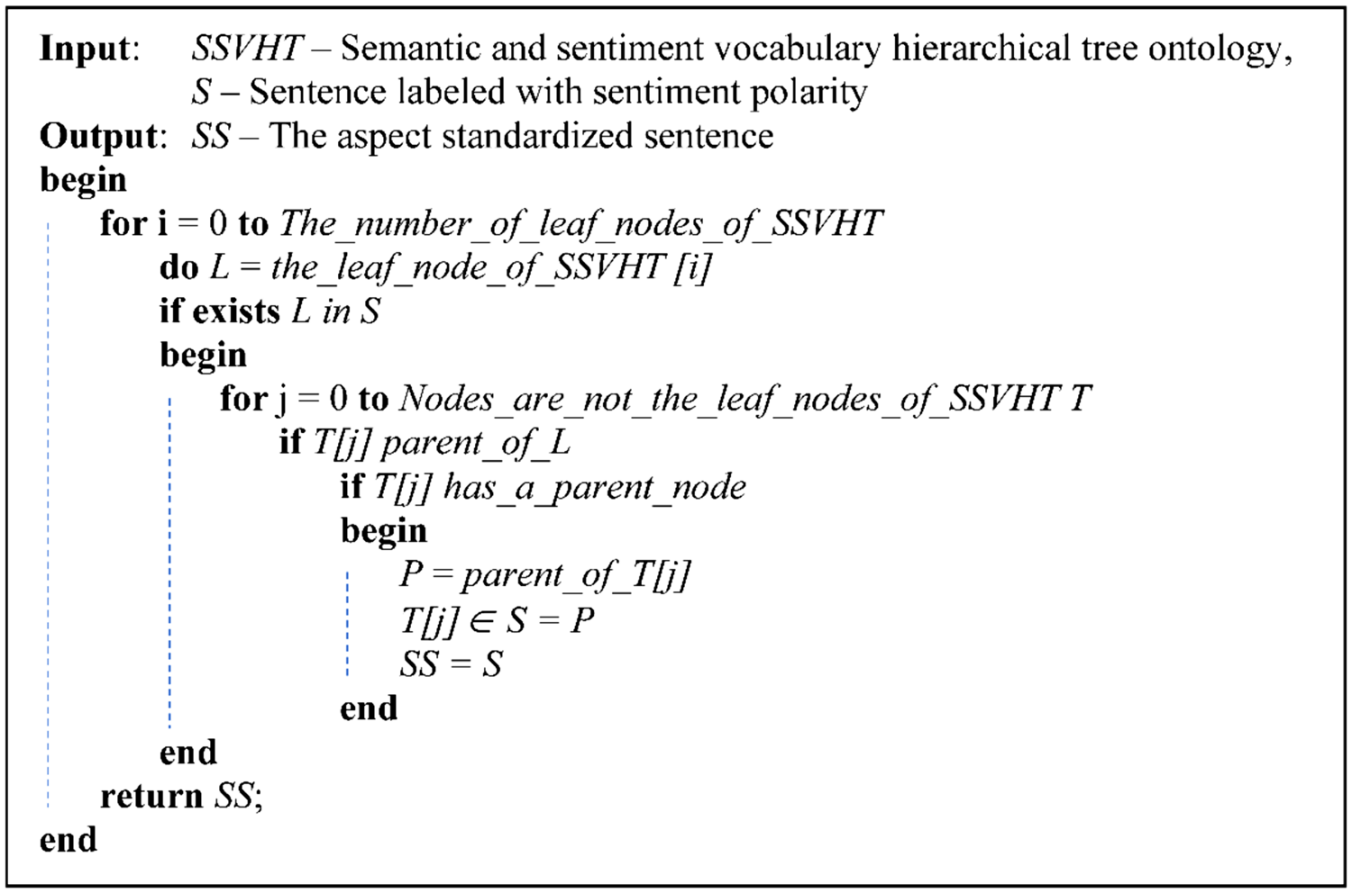
Algorithm of aspect standardization for the sentence.

This aspect standardization process involves replacing the standard aspect equivalent to the word meaning the standard aspect. Each sentence is aspect analyzed, creating a copy and identifying the word indicating the aspect in the sentence. This word is used, which relies on the SSVHT ontology to find the standard aspect term if it is not a standard aspect term. We used the standard aspect word instead of the word. This step is omitted if the sentence contains only a standard aspect. The aspect standardization algorithm is presented in Fig. 3.

We set *S* = {*s*_*1*_*, s*_*2*_*,…, s*_*k*_} as the set of *k* words indicating the standard aspects.

We set *E*_*1*_ = {*e*_*11*_*, e*_*12*_*,…, e*_*1m*_} as the set of *m* words indicating the standard aspect-equivalent word *s*_*1*_ in the corpus. Similarly, we set *E*_*k*_ = {*e*_*k1*_*, e*_*k2*_*,… e*_*km′*_} as the set of *m′* words indicating the standard aspect equivalent word *s*_*k*_ in the corpus, and so on.

We set *V*_*1*_ = {*v*_*11*_*, v*_*12*_*,…, v*_*1n*_} as the set of *n* words related to word *e*_*11*_ in the corpus. Similarly, we set *V*_*m*_ = {*v*_*m1*_*, *v_m2_*,…, v*_*mn'*_} as the set of *n′* words related to the word *e*_*1m*_ in the corpus, and so on.

The aspect standardization process creates a relationship between the standard aspect word with other words, that is, the standard aspect equivalent words. Thus, the vocabulary set related to the standard aspect *s*_*1*_ is *V*, as given in Eq. (2):

2 $$ V = V_{1} \cup V_{2} \cup ... \cup V_{m} = \bigcup\limits_{i = 1}^{m} {V_{i} } $$

then

3 $$ \left| V \right| \, \ge \, \left| {V_{i} } \right|,\;\forall i \in \left[ {1;m} \right] $$

Using Eq. (3), the vocabulary set related to standard aspect *s*_*1*_ is estimated, which is at least as large as the set of words that each of the words is similar to or approximately related to. The same applies to other standard aspect words.

The expansion of words with a related vocabulary is the same as the expansion of the corpus containing those words.

#### Generalizing aspect review sentences

The aspect element and sentiment word used to review the aspect are two important components of opinion. In this study, we extracted sentimental words (determined by the SSVHT ontology) and modifiers for sentimental words in sentences to show the relationship between them and the standard aspect component in opinions. This relationship was built using sentence samples 4, 5, and 6 to create a generalized review for opinion.

4 $$ {\text{N }} + {\text{ A}} $$

5 $$ {\text{N }} + {\text{ R}} + {\text{A}} $$

6 $$ {\text{N }} + {\text{ A}} + {\text{R}} $$

where N: the noun indicating the aspect of the car, R: adverb or adverbs, A: sentiment complements (adjectives or verbs).

The following example illustrates the process of standardizing an opinion:

Example 2.6

S1 = “Theo mình thấy **ghế da** em này ***khá ngon*** trong phân khúc đấy chứ” (*In my opinion, this* leather** chair** *is ****rather delectable**** in this car segment.*)

In Example 2.6, the S1 opinion includes only one sentence with the “leather seat” phrase as a component of the car, and the “quite good” adjectival phrase is used to evaluate the “leather chair” component. In the SSVHT ontology, the standard aspect layer element related to the “leather seat” is the “interior.” To highlight the relationship between the element in the standard aspect equivalent class and the standard aspect layer of the ontology, we created a copy of sentence S1, in which the word “leather seat” is replaced by the word “interior” as in sentence S2 in Example 2.7.

Example 2.7

S2 = “Theo mình thấy **nội thất** em này ***khá ngon*** trong phân khúc đấy chứ” (*In my opinion, this* **interior** *is ****rather delectable**** in this car segment.*)

We express the relationship between the aspect and sentiment words using sentence samples 2.4, 2.5, or 2.6, similar to sentence S3 in Example 2.8, to generalize the content of the aspect review sentence.

Example 2.8

S3 = “nội thất khá ngon” (The interior is quite delectable).

**Figure 4:**
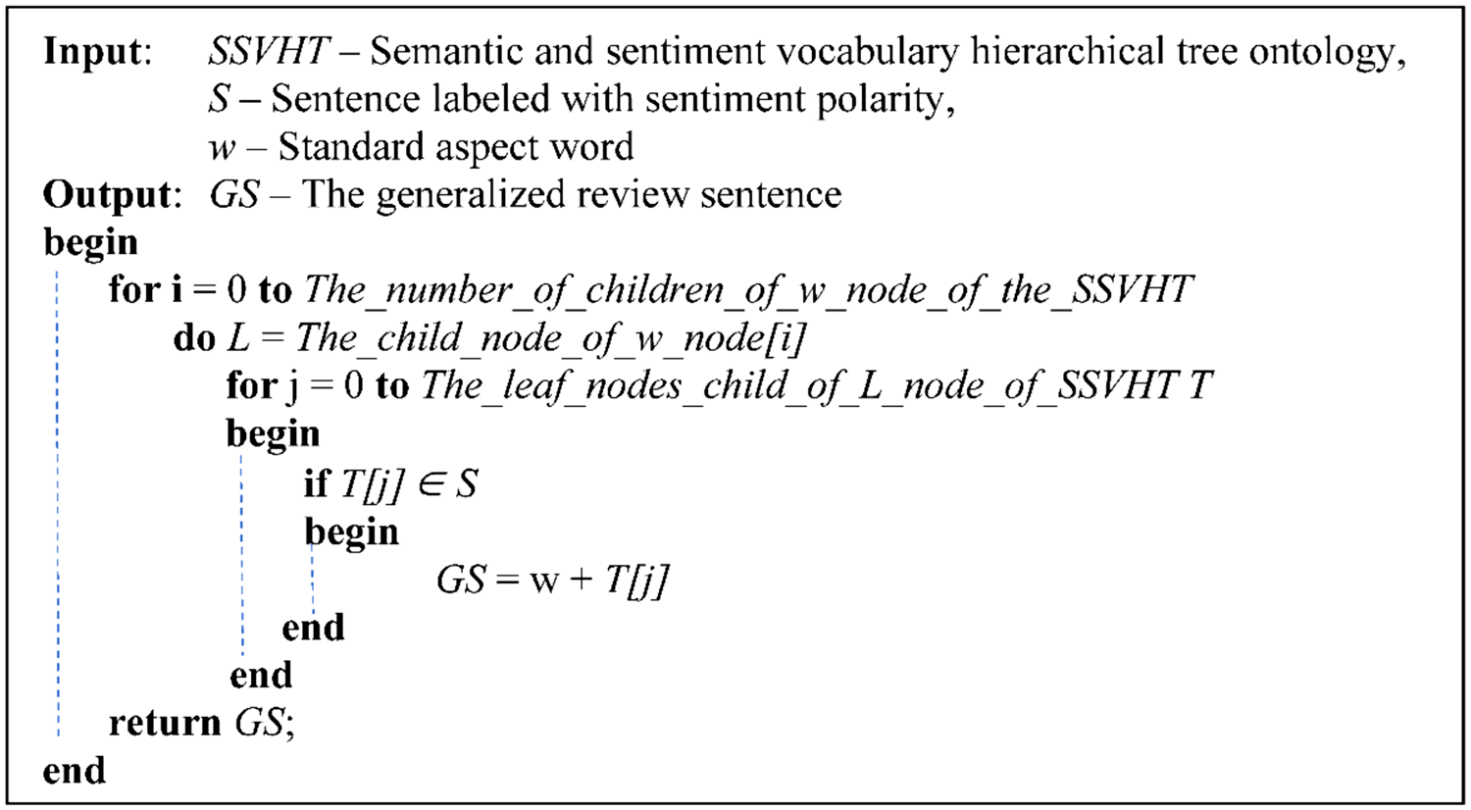
Generalization of opinions by the algorithm.

The algorithm generalization of opinions is presented in Fig. 4.

Set *X*_*1*_ = {*x*_*11*_*, x*_*21*_*, …, x*_*q1*_} is the set of *q* sentiment words that are context-related to the standard aspect equivalent word *e*_*11*_, and set $X_{1}^{^{\prime}} = \{ x_{11}^{\prime} ,x_{21}^{\prime} ,\ldots,x_{p1}^{\prime} \}$ is the set of *p* sentiment words that are context-related to the standard aspect equivalent word *e*_*11*_ but limited by technical factors (the window size component of the Gensim library by Rehurek and Sojka^29^ when building word2vec) that arise when calculating word2vec. Similarly, set *X*_*m*_ = {*x*_*1m*_*, x*_*2m*_*,…, x*_*q'm*_} is the set of *q'* sentiment words that are context-related sentiment words with the standard aspect equivalent word *e*_*1m*_, and set $X_{m}^{^{\prime}} = \{ x_{1m}^{\prime} ,x_{2m}^{\prime} ,\ldots,x_{p^{\prime}m}^{\prime} \}$ is the set of *p'* sentiment words context-related to the standard aspect equivalent word *e*_*1m*_ but limited by technical factors.

The aspect standardization and generalization of the aspect review sentence create the set of sentiment word *X* related to the context with the standard aspect word *s*_*1*_. X is defined as Eq. (7):

7 $$ X = X_{1} \cup X^{\prime}_{1} \cup ... \cup X_{m} \cup X^{\prime}_{m} = \bigcup\limits_{i = 1}^{m} {X_{i} } \cup \bigcup\limits_{i = 1}^{m} {X^{\prime}_{i} } $$

then

8 $$ \left| X \right| \, \ge \, \left| {X_{i} } \right|,\;\;\forall \in \left[ {1; \, m} \right] $$

Based on Eq. (8), the standard aspect word *s*_*1*_ has a contextual relationship with a set of sentiment words that is at least as large as that of sentiment words with contextual relationships, in which each word is either exactly or approximately the same. This also applies to other standard aspects.

Thus, the standardized aspect process makes standard aspect words related to a large set of sentiment words. This is equivalent to expanding the corpus containing the words.

#### Embedding the ontology into the corpus

**Figure 5:**
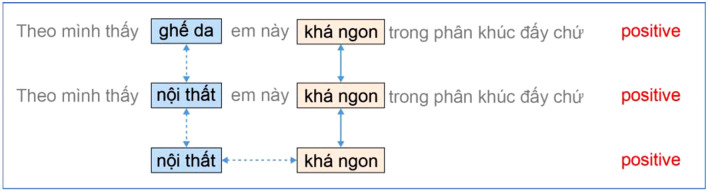
Image showing the relationship between the correlation aspect, the standard aspect, and the sentiment word.

For each aspect assessment sentence in the raw corpus, as with S1 in the above section, after aspect standardization and generalization, two sentences, such as S2 and S3 in the above section, represent the relationship between the standard aspect word and the sentiment word in the SSVHT ontology. Sentences S2 and S3 were added to the corpus. Figure 5 illustrates the relationship between the sentimental and semantic components between S1, S2, and S3.

The corpus is created from the KPRO method with five components:

- Opinion dataset collected from forums or social networks (opinion data: OD). This dataset has 2994 samples.
- The aspect standardized opinion dataset from OD is the aspect standardized data (ASD). This dataset has 2994 samples.
- The dataset of aspect-separated sentences from the OD's sentences by the method presented in the “Experiment” section involves the aspect sentences (AS). This dataset has 9059 samples.
- The AS dataset is aspect-standardized by the method presented in the “Experiment” section, called aspect standardized sentences (ASS). This dataset has 6655 samples.
- The opinion dataset is generated from the combination of an aspect term, and a sentimental term is called generated data (GD) by the patterns (4), (5), and (6). This dataset has 22,829 samples.

These datasets have three sentiment polarities as positive, neutral, and negative. The OD and ASD datasets included opinions with two or more sentences only. The AS and ASS datasets contained opinions with one sentence.

## Experiment

This study built a test scenario to evaluate the effectiveness of the KPRO method at the word level, sentiment classification at the aspect level, and sentiment classification at the document level.

### The algorithms used for experiments

In this section, the authors briefly describe the experimental methods mentioned in the introduction section.

#### word2vec^4^

**Figure 6:**
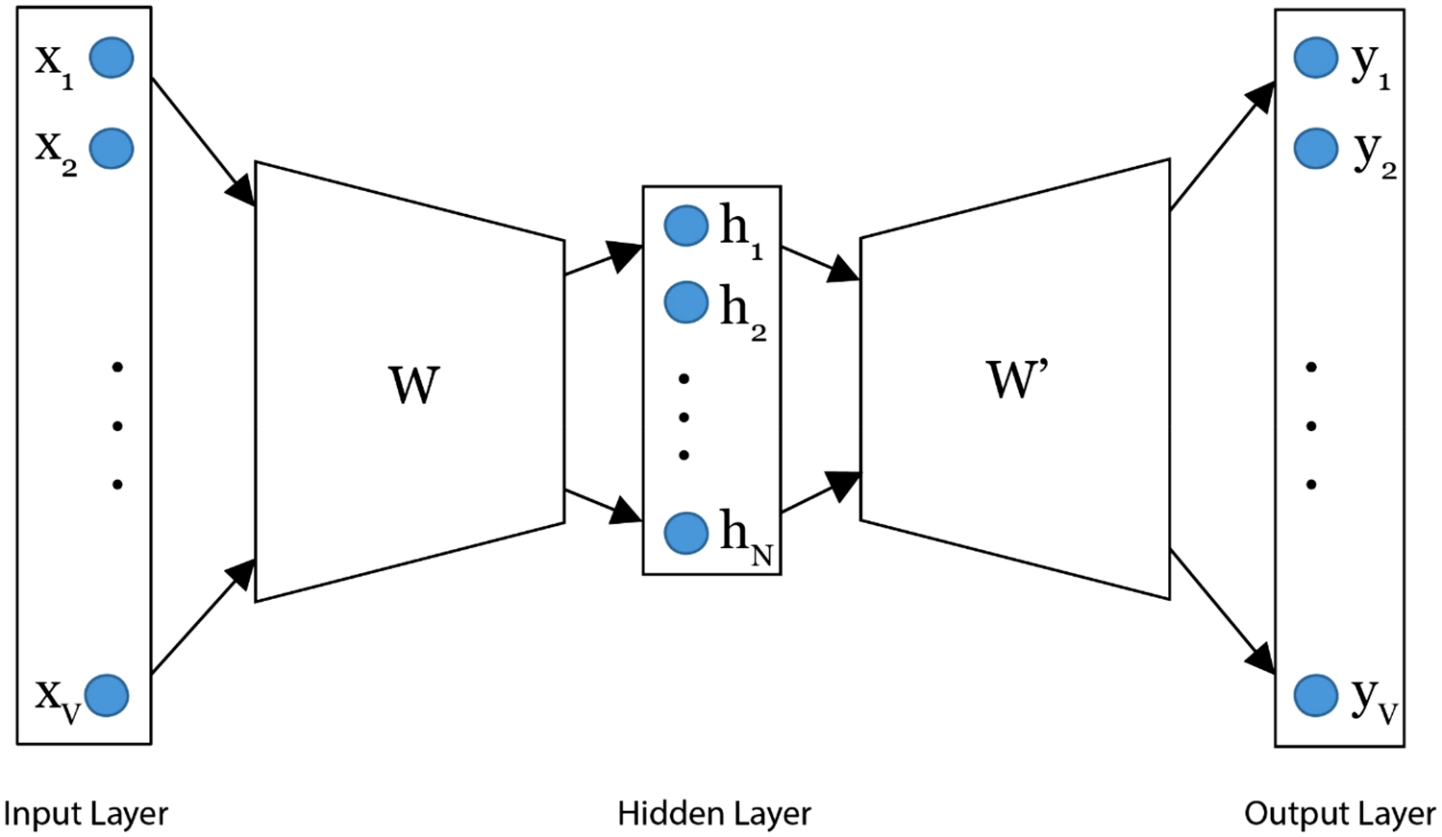
A continuous bag-of-words model.

word2vec uses a neural network model to learn word links from a corpus. It represents each word, along with its semantic features, using a real number vector. There are two models for implementing word2vec: continuous bag-of-words (CBOW) and skip-gram^30^. The CBOW model predicts the target word from words in the same context. The skip-gram model uses the context words to predict the target words. Figure 6 illustrates a simple CBOW model.

The CBOW model calculates the negative logarithmic probability of a word (*w*_*o*_) relative to the context (*w*_*i*_), following Eq. (9):

9 $$ - logP(w_{o} |w_{i} ) $$

The expression *P(w*_*o*_*|w*_*i*_*)* is calculated using Eq. (10):

10 $$ P({w_o}|{w_i}) = \frac{{\exp (v{{_{{w_o}}^{\prime}}^T}{v_{{w_i}}})}}{{\sum\nolimits_{v = 1}^V {\exp \left(v{{_{{w_v}}^{\prime}}^T}{v_{{w_i}}}\right)} }}$$

where *V* is the number of the input layer vocabulary, *N* is the dimension of the hidden layer, *W* is the weighting matrix between the input and hidden layers, and the size of *W* is *V* × *N*. Each row of *W* is a vector *v*_*w*_ with dimension number *N*, and *v*_*w*_ is the input vector of *w*, *W′* is the weight matrix between the hidden layer and the output layer of size *N* × *V*

$$W^{\prime} = \left( \begin{array}{l}
v_{{w_1}}^{{\prime^T}}\\
v_{{w_2}}^{{\prime^T}}\\
\ldots \\
v_{{w_V}}^{{\prime^T}}
\end{array} \right)$$ $$v^{\prime}_{w}$$ is the output vector of word *w*.

The skip-gram model is similar to the CBOW model, except that it takes one word as input and predicts all other output words.

#### tf-idf^6^

This method considers the frequency of each word as a feature of the text. Word importance in a text is represented by the value calculated based on the statistics of the word appearing in a text and a text set. The characteristics of the text according to the TF-IDF are determined by the term frequency (TF) and inverse document frequency (IDF) according to Eqs. (11), (12), and (13).

11 $$ TF(t,d) = \frac{n(t,d)}{N} $$

12 $$ IDF(t,D) = \log \left( {\frac{|D|}{{DF(t)}}} \right) $$

13 $$ TF - IDF(t,d,D) = TF(t,d) \times IDF(t,D) $$

where *n(t, d)* is the number of times term *t* appears in document *d*, *N*: number of words in document *d*, |*D*|: number of documents in the training dataset, *DF(t)*: number of documents where the term *t* appears.

#### CNN^12^

Each CNN contains a word embedding layer, convolutional layer, pooling layer, and fully connected layer. The word embedding layer consists of matrices of size *n* × *k*, representing sentences with *n* words; each word represents a *k* dimensional vector. The convolutional layer uses convolutional calculus to process data by sliding the fixed-size slide window (also called the kernel) on the input data matrix to obtain refined results. The pooling layer combines the result vectors of the convolutional layer and retains the vectors that matter the most. The fully connected layer is a traditional neural network that uses the remaining vectors in the upper layers as input to produce the final result through training.

#### LSTM^31^

The LSTM method is a deep learning method used for regression analysis (recurrent neural networks (RNN)) and is suitable for processing information sequentially. In RNN, the connecting neurons are cyclical and directed. The output of a node is dependent on all previous nodes' inputs and remembers information. An LSTM unit consists of a cell, an input gate, an output gate, and a forget gate. The cell remembers values at random intervals, and the three gates regulate the flow of information in/out of the cell.

#### Bi-LSTM^31^

The Bi-LSTM model is based on the idea that the output at a moment can depend not only on the previous elements in the series but also on the next elements in the chain. This model comprises two sets of stacked reverse RNNs. An input processor is in the initial order, and one RNN handles the reverse input sequence. The output is then computed based on the hidden states of both the RNNs.

#### BERT^7^

The BERT method uses a bidirectional transformer network developed by Ashish et al.^32^ to pre-train a language model using a large corpus and then refines the pre-trained model on other tasks. In the WordPiece method, BERT is used for data processing, separate words with special characters ##, using tokens [CLS], [SEP] to distinguish the beginning of the string or the beginning of the sentence, token [MASK] uses to conceal words. If there is a pair of sentences combined into a string, they are marked with different segments after each token [SEP]. Sentences or pairs of sentences are represented as a series of words.

The BERT input representation consists of position embeddings, segment embeddings, token embeddings, and input layers. Data for the input layer is obtained by summing the vocabulary of token embedding, sentence embedding, and the transformer position embedding element for a given vocabulary. For the classification problem, the first word of the string is identified by the token (CLS), and a fully connected layer is connected in place (CLS) of the last encryption layer.

#### SVM^33^

SVM is a supervised machine-learning algorithm introduced by Vladimir N. Vapnik in 1995. The basic principle of SVM is that it finds a hyperplane to separate the data. This hyperplane divides the space into different domains containing one type of data. The distance from the nearest point to the hyperplane (called the margin) is as large as possible so that the classification error is minimal. This method can be classified as nonlinear or linear. This method can efficiently cope with high-dimensional feature spaces.

#### Naïve Bayes^34^

Naïve Bayes is a classification method based on probability calculation using Bayes’ theorem. This method is a supervised learning method. It calculates the random probability of event y given event x using Eq. (14):

14 $$ P(y/x) = \frac{P(x/y).P(y)}{{P(x)}} $$

The naïve Bayes method is computed based on the assumption of probability independence between attributes.

#### CNN-LSTM and CNN-Bi-LSTM

The CNN convolutional layer creates a feature vector for the object. The number of feature vectors is equal to the number of filters used in the convolution process. Each layer's best feature values are selected to derive the opinion's most important feature in the pooling layer. The feature vectors processed by the fully connected layer create a set of parameters at the CNN output. LSTM and Bi-LSTM models use CNN output parameters to perform sentiment classification.

### Experimental design

In this study, we studied numerous aspects of the corpus at the word, aspect, and document level to evaluate its effectiveness. These experiments are implemented when the data are not processed by the KPRO method, and in this case, it is processed by this method.

#### Corpus

Based on the testing criteria outlined in the above section, the corpus of this study was divided into the following parts:

Data for testing: randomized by the random number generation functions of the MS SQL Server and had about 30% data. There were two test datasets:

- Dataset used to test the aspect-level sentiment classification (ALT3): extracted from the AS dataset: 3001 (sentences).
  There are two test data types: raw data RALT (raw ALT) and aspect-standardized data SALT (standardized ALT). The RALT dataset was used to evaluate the aspect-level sentiment classification performance of the algorithm when trained on a dataset that was not processed by the KPRO method. The SALT dataset is the sentence level of the RALT dataset, which is standardized in terms of aspect. The SALT dataset was used to evaluate the algorithms' aspect-level sentiment classification performance when they were trained on the dataset processed by the KPRO method.
- Dataset used to test the document-level sentiment classification (opinion-level test—OLT): extracted from the OD dataset: 901 (opinions).
  Similar to the ALT aspect-level sentiment classification test dataset, the document-level sentimental classification test dataset also has a raw (ROLT) dataset which is used to evaluate the algorithms' document-level sentiment classification performance when the algorithm is trained on datasets not processed by the KPRO method and an aspect-standardized standardized OLT (SOLT) dataset to evaluate the algorithms' document-level sentiment classification performance when the algorithm is trained on the dataset processed by the KPRO method.
  Both ALT and OLT have three sentiment polarities: positive, neutral, and negative. When using ALT and OLT to test sentiment classification with positive and negative polarity, samples with neutral sentiment polarity are omitted.
- Training data (exclude samples of ALT and OLT dataset):
  -  The raw aspect-level training dataset with three sentiment polarities as positive, negative, and neutral, called AS3 (Aspect Sentences 3).
    AS3 = AS \ ALT = 6057 (sentences).
  -  The aspect-level training dataset was processed by the KPRO method with three sentiment polarities: positive, negative, and neutral, and was called aspect-level sentences 3 (SAS3), which included the raw dataset, aspect-level standardized dataset, and self-generated dataset.
    SAS3 = AS3 ∪ ASS ∪ GD = 32,539 (sentences).
  -  The raw aspect-level training dataset with two sentiment polarities called aspect sentences 2 (AS2) included samples of the AS3 dataset, excluding samples with neutral polarity: 4204 (sentences).
  -  The aspect-level training dataset was processed by the KPRO method with two sentiment polarities—positive and negative—and was called set of aspect-level sentences 2 (SAS2); it included samples of the SAS3 dataset excluding samples with neutral polarity: 25,035 (sentences).
  -   The raw document-level training dataset with three sentiment polarities—positive, negative, and neutral—called opinion data 3 (OD3) included samples of the OD dataset excluding the opinion of the OLT dataset:
    OD3 = OD\OLT = 2093 (opinions).
  -  The raw document-level training dataset with two sentiment polarities—positive and negative—called opinion data 2 (OD2) included samples of OD3 excluding samples with a neutral sentiment polarity: 1606 (opinions).
  -  The aspect-standardized document-level training dataset with three sentiment polarities—positive, negative, and neutral—called aspect standardized data 3 (ASD3) included opinion samples from the ASD dataset, excluding samples of the OLT dataset:
    ASD3 = ASD\OLT = 2093 (opinions).
  -  The aspect-level standardized training dataset with two sentiment polarities—positive and negative—called aspect standardized data 2 (ASD2) included samples of ASD3 excluding samples with neutral polarity: 1606 (opinions).
  -  The document-level training dataset included the raw aspect-level AS dataset with the raw opinion OD dataset and was called raw combined corpus 3 (RCC3) with three sentiment polarities: positive, negative, and neutral.
    RCC3 = AS3 ∪ OD3 =  8150 (samples).
  -  The raw document-level training dataset combined the raw aspect-level AS dataset with the raw opinion OD dataset and was called raw combined corpus 2 (RCC2). The two sentiment polarities were positive and negative.
    RCC2 = AS2 ∪ OD2 =  5792 (samples).
  -  The full combined corpus 3 (FCC3) dataset include all training datasets with three sentiment polarities:
    FCC3 = SAS3 ∪ OD3 ∪ ASD3 = 36,725 (samples).
  -  The full combined corpus (FCC2) dataset included all training datasets with two sentiment polarities.
    FCC2 = SAS2 ∪ OD2 ∪ ASD2 = 28,247 (samples).
    The number of datasets used for training and testing to evaluate the effectiveness of the proposed KPRO method is shown in Table 2.

**Table 2:** Details of the components of the corpus in Vietnamese.

Dataset	Quantity
AS3	6057
AS2	4204
SAS3	32,539
SAS2	25,035
OD3	2093
OD2	1696
ASD3	2093
ASD2	1606
RCC3	8150
RCC2	5792
FCC3	36,725
FCC2	28,247
ALT	3001
	Negative: 949
	Positive: 1039
	Neutral: 1013
OLT	901
	Negative: 350
	Positive: 403
	Neutral: 148

#### Word-level experiment

The calculation model of word2vec can identify the near and far correlations of semantics or roles between words in the corpus after processing. This uses the word2vec tool to search for terms related to a given word. It is based on the semantic elements of related words found in word2vec to determine the representation degree of the corpus topic.

This study selected a general configuration of the word2vec tool to handle different datasets and evaluate the effectiveness of the proposed method. The configuration parameters of the word2vec tool have numbers as below.

- Size: 300
- Window: 5
- Min_count: 2
- Worker: 10
- Epoch: 200
- Algorithm: CBOW

We analyzed the words similar and related to 10 words in the popular meaning sentiment and 10 words in the popular meaning aspect. The number of search words was 30.

- 10 words of sentiment: đẹp (*beautiful*), xấu (*ugly*), ngon (*delectable*), đắt (*expensive*), rẻ (*cheap*), sang (*luxurious*), yếu (*weak*), mạnh (*strong*), tốt (*good*), êm (*pillowy*).
- 10 words of aspect: nội_thất (*interior*), máy_lạnh (*air-conditioning*), vô_lăng (*steering wheel*), kiểu_dáng (*style*), ghế (*chair*), cửa (*door*), đèn (*lamp*), máy (*machine*), ngoại_thất (*exterior*), phanh (*brake*).

#### Aspect-level sentiment classification

We used the aspect analysis datasets introduced in the above section. Datasets AS3, AS2, SAS3, and SAS2 were used to train the machine learning algorithms for this task. These datasets are described in the above section. In this case, the test data were RALT and SALT.

#### Document-level sentiment classification

Although the KPRO method is based on aspect-based analysis, this study also experiments with document-level sentiment classification. Datasets OD3, OD2, ASD3, ASD2, RCC3, RCC2, FCC3, and FCC2 were used to train the machine learning algorithms. These datasets are described in the above section. In this case, the test data were ROLT and SOLT.

### Experimental setting

The basic experiment configurations of the algorithms used in this study are presented in Table 3. This study used the Keras library to model the deep learning algorithms, as mentioned in the “Introduction” section. SVM and naïve Bayes algorithms were implemented using the Sklearn library.

**Table 3:** Setting of models.

Parameter	CNN	LSTM	Bi-LSTM	BERT-base	SVM	Naïve Bayes
Epoch	500	500	500	500	–	–
Pre-trained model	–	–	–	bert_uncased_L-12_H-768_A-12/1	–	–
Activation/Kernel	sigmoid	sigmoid	ReLU		rbf	–
Number of filters	300	300	300			
Dropout	0.3	0.3	0.5		–	–
Batch size	512	512	512	32	–	–
Feature process	word2vec	word2vec	word2vec	–	tf-idf	tf-idf

The hardware configuration used to run the algorithms with the parameters in Table 3 is as follows:

- CPU: Intel Core i7 8700
- RAM: 64 GB
- GPU: RTX 2080 Ti and Tesla K80.
- SSD: 500 GB NVMe PCIe
- Operating system: Ubuntu 20.04 LTS
- Programming language: Python 3.7

The CNN, LSTM, CNN_LSTM, CNN_Bi-LSTM, SVM, and Naive Bayes models only use RTX 2080 Ti card. The BERT and Bi-LSTM model must use both cards present in the computer. The BERT model uses the Horovod^35^ library to run multi GPU. Each model performs three runs to obtain three results. The average of these three results is the final result.

### Experimental results

The test results according to the construction scenario above are presented in Tables 4, 5, 6, 7, and 8.

**Table 4:** The entity identity degree is closely related to the aspect or sentiment of the target word using the word2vec tool for datasets AS3, SAS3, RCC3, and FCC3.

Feature	AS3 (%)	SAS3 (%)	RCC3 (%)	FCC3 (%)
Aspect terms	83.33	93.00	91.00	94.67
Sentiment term	89.67	96.00	85.67	96.33
Overall	86.50	94.50	83.33	95.50

**Table 5:** Accuracy (%) of the algorithms when trained with the AS3 and SAS3 datasets.

Model	AS3	SAS3	Difference (%)
LSTM	78.12	80.11	1.99
CNN	79.66	81.98	2.32
Bi-LSTM	77.69	79.88	2.19
CNN_LSTM	76.52	**84.04**	**7.52**
CNN_Bi-LSTM	76.59	79.81	3.22
SVM	77.32	78.68	1.36
Naïve Bayes	73.08	76.13	3.05

Significant values are in bold.

**Table 6:** Accuracy (%) of the algorithms trained with the AS2 and SAS2 datasets and the difference (%) of the accuracy in these two cases.

Model	AS2	SAS2	Difference (%)
LSTM	89.55	91.17	1.62
CNN	90.35	**91.88**	1.53
Bi-LSTM	89.09	90.82	**1.73**
CNN_LSTM	90.45	91.73	1.28
CNN_Bi-LSTM	90.20	91.38	1.18
SVM	89.44	88.80	− 0.64
Naïve Bayes	87.17	86.89	− 0.28
BERT-base	52.22	90.36	38.14

Significant values are in bold.

**Table 7:** Accuracy (%) for document-level sentiment classification of algorithms achieved when trained with datasets OD3, ASD3, RCC3, and FCC3.

Model	OD3	ASD3	RCC3^I^	FCC3^II^	RCC3^III^	FCC3^IV^
LSTM	57.89	61.00	73.14	81.24	73.14	80.13
CNN	63.22	64.56	71.81	80.69	72.03	81.02
Bi-LSTM	61.27	63.82	73.58	80.91	73.25	80.47
CNN_LSTM	56.00	57.44	74.81	80.24	73.81	79.80
CNN_Bi-LSTM	57.44	56.33	72.59	79.58	73.14	81.35
SVM	62.11	64.22	–	–	69.15	70.81
Naïve Bayes	59.93	63.04	–	–	68.18	63.71

**Table 8:** The accuracy (%) achieved by the algorithms in document-level sentiment classification when trained with the OD2, ASD2, RCC2, and FCC2 datasets.

Model	OD2	ASD2	RCC2^I^	FCC2^II^	RCC2^III^	FCC2^IV^
LSTM	67.38	76.66	82.53	88.07	81.80	88.07
CNN	71.39	76.04	83.15	88.81	83.39	90.10
Bi-LSTM	68.26	78.29	82.90	87.21	81.80	88.31
CNN_LSTM	68.01	77.54	82.16	88.19	82.90	89.71
CNN_Bi-LSTM	65.37	76.66	82.66	88.07	82.66	89.94
SVM	72.40	71.14	–	–	81.54	86.35
Naïve Bayes	69.26	69.01	–	–	79.34	80.81
BERT-base	62.40	73.88	50.00	82.16	–	–

#### Word-level experiment

The measured values determining the similar or related words for the 20 words in the datasets in Table 2 are presented in Table 4.

The data in Table 4 show that the ability to find approximate words in the datasets processed by the KPRO method (SAS3 and FCC3) is much better than its ability to find those in raw datasets (AS3 and RCC3). This will be the basis for deep learning methods to understand language, thereby improving deep learning methods used for sentiment classification and other problems.

#### Aspect-level sentiment classification experiment

In this experiment, the training datasets are AS3, AS2, SAS3, and SAS2 introduced above.

- Sentiment analysis for the dataset with three sentiment polarities
  The data were labeled with positive, negative, and neutral sentiment polarities. The best results of the other models in the experiment are presented in Table 5.
  The following are some notable points from the results in Table 5:
  - All deep learning models used in this experiment were more accurate when trained with datasets processed by the KPRO method (SAS3 dataset) than when trained with the raw dataset (AS3 dataset). The lowest difference in accuracy was 1.99% for the LSTM model. The highest difference in accuracy was 7.52% for the CNN_LSTM model. The difference in the two data cases shown in Table 4 is a very significant improvement, even though the AS3 dataset is not really raw data but has been aspect-split processing.
  - The SVM and naïve Bayes algorithms are less effective than deep learning models when trained using the SAS3 dataset. With the AS3 dataset, the difference is trivial.
  - It can be said that repeating the sentence in which only the word mean aspect (normalized aspect) of the KPRO method is changes does not make sense when considering word frequency.
- Sentiment analysis for the dataset with two sentiment polarities
  The training data were AS2 and SAS2. These datasets included only positive and negative labels. The best results for each model in this experiment are presented in Table 6.
  - The accuracy achieved by all the methods is much higher than that of the opinion classification with three sentiment polarities (Table 5). Neural network-based methods trained by datasets processed by the KPRO method (SAS2) are mostly over 91% (except for the Bi-LSTM method) and higher when trained by the datasets not processed by this method (AS2). The difference in the test accuracies of the two data types was approximately 1.5%. This difference was very significant at over 90% accuracy. The AS2 dataset was aspect split processed as well.
  - The difference in the accuracies for the two data cases in this experiment is significant when tested with the BERT method. With the SAS2 dataset, the BERT-base method achieved an accuracy of 90.36%, which is equivalent to other deep learning methods. However, the accuracy of 52.22% by BERT when testing with a raw corpus was too low. Thus, it can be said that the KPRO data processing method performed well. With the same amount of information, an appropriate data processing method can increase the efficiency of a deep learning method that requires a large amount of data and hardware, such as BERT.

#### Document-level sentiment classification

In this experiment, the training datasets were OD3, OD2, ASD3, ASD2, RCC3, RCC2, FCC3, and FCC2, as introduced in the above section.

- Sentiment analysis for the dataset with three sentiment polarities
  The test data for this purpose were obtained from the OLT dataset. The best results for each model in this experiment are presented in Table 7.
  Note:
  - RCC3^I^: Algorithms trained with dataset RCC3; word2vec built from dataset AS3.
  - FCC3^II^: Algorithms trained with dataset FCC3; word2vec built from dataset SAS3.
  - RCC3^III^: Algorithms trained with dataset RCC3; word2vec built from dataset RCC3.
  - FCC3^IV^: Algorithms trained with dataset FCC3; word2vec built from dataset FCC3.
  The following are some notable points from the results in Table 7:
  - The effectiveness of the KPRO method in this experiment is apparent. The accuracy of sentiment classification of deep learning algorithms when trained with the KPRO (FCC3)-processed data is significantly different from that of raw data (OD3). The best accuracy improvement was up to 23.91% (CNN_Bi-LSTM model).
  - Algorithms were trained using datasets comprising both aspect- and document-level data processed using methods such as the KPRO method; however, the data were not processed by following the steps mentioned in the above section (RCC3). Further, the accuracy was also significantly lower than that observed when the model was trained using the dataset processed using the KPRO method (FCC3).
  - There was no major difference in document-level sentiment classification when experiments were conducted on only one training dataset (RCC3 or FCC3). However, the word embeddings were generated using word2vec computed on an aspect analysis dataset (RCC3^I^ và FCC3^II^), compared to the word embeddings that were created using word2vec computed from a dataset comprising aspect-level and document-level data (RCC3^III^ và FCC3^IV^).
  - Similar to the aspect-level sentiment classification experiment, the SVM and naïve Bayes methods are not as effective as the deep learning models.
- Sentiment analysis for the dataset with two sentiment polarities
  The test data for this purpose is the OLT dataset, which omits comments labeled neutral. The best results for each model in this experiment are presented in Table 8.
  Note:
  - RCC2I: Algorithm trained by the RCC2 dataset; word2vec built from the AS2 dataset.
  - FCC2II: Algorithm trained by the FCC2 dataset; word2vec built from the SAS2 dataset.
  - RCC2III: Algorithm trained by the RCC2 dataset; word2vec built from the RCC2 dataset.
  - FCC2IV: Algorithm trained by the FCC2 dataset; word2vec built from the FCC2 dataset.
  The following are some notable results in Table 8:
  - Similar to the sentiment classification with the dataset with three polarities (Table 7), the effectiveness of the KPRO method in this experiment is apparent. The accuracy of the sentiment classification of deep learning algorithms when trained with the KPRO (FCC2)-processed data is significantly different from that of sentiment classification when trained with raw data (OD2). The best accuracy improvement was up to 24.57% (CNN_Bi-LSTM model). The algorithms trained by the RCC2 dataset were also much less accurate than those trained using the FCC2 dataset.
  - The accuracy achieved by the SVM and naïve Bayes algorithms was not as good as that achieved by the deep learning algorithms. In particular, the performance of the naïve Bayes method was the worst. This shows that the KPRO method does not make a difference in terms of word frequency, but rather creates a relationship between words to represent knowledge in the corpus and ontology.
  - In these experiments, the BERT method did not yield better results than the other deep learning methods. The best accuracy was 82.16%. The difference of 19.76% when FCC2 and OD2 are used to train BERT shows that KPRO is highly effective.

## Discussion

The results of the document-level sentiment classification experiment with raw datasets such as OD2 and OD3 achieved low accuracy (the accuracy achieved by algorithms is less than 64% with raw data, and less than 70% with processed data except for CNN), and there is a significant difference between the algorithms, as shown in Tables 7 and 8. The LSTM-based sequence data processing method had difficulty dealing with long texts. The LSTM-based models achieved a lower accuracy than the CNN model (3% compared to 6% on the dataset with two sentiment polarities and 2% to 7% on the dataset with three sentiment polarities). However, in experiments on the corpus that embedded knowledge from the ontology of the KPRO method, the algorithms produce an accuracy of over 24% (in the case of CNN_Bi-LSTM model in data testing has two sentiment polarities). The CNN algorithm with high-level feature extraction achieved the best results in the experiments, although the LSTM-based models achieved the same accuracy as the CNN model. Thus, the KPRO method has helped the LSTM-based models achieve high accuracy on data with long-distance contextual relations. The BERT model failed to achieve as high an accuracy as other deep learning models, but the improvement in its accuracy was the most dramatic (over 32%).

With aspect-level sentiment classification, with the dataset carefully processed, the algorithms can achieve high accuracy even with data not processed by the KPRO method. Since the aspect-level data do not consist of particularly long sentences, the sequential processing-based models achieved the same accuracy as the CNN model. Under such conditions, the KPRO method also helps the models improve by approximately 3% when experimenting with the three-sentiment polarities dataset and by approximately 2% with the two-sentiment polarities dataset.

This research uses the basic model used for deep learning algorithms, as opposed to introducing a new model, in order to rule out any risk of the improvement factor biasing the results towards the processing direction of the KPRO method or the opposite. Therefore, the experimentally observed performance improvement is the effect of the KPRO method. However, a limitation in the document-level sentiment classification is the small size of the OD2 dataset. The size of the OD2 dataset is much reduced compared with OD3 after omitting the samples with neutral polarity, leaving only 1700 samples (see Table 2). However, with the high confidence data set preparation, the algorithms still achieve an accuracy of approximately 70%, except for the BERT algorithm, which uses a characteristic calculation method based on the WordPiece method.

## Empirical evaluation

In document-level sentiment classification, when the algorithm is trained with the document-level dataset that combines aspect-level data and document-level data (FCC2 and FCC3 datasets) and processed by the KPRO method, the accuracy obtained is much better than that obtained by training with only with document-level datasets (ASD2 and ASD3 datasets), even though the aspect-level data are information drawn from opinions. Meanwhile, if we perform the same process using raw data (RCC2, RCC3, OD2, and OD3 datasets), the accuracy of the deep learning algorithms in sentiment analysis is much lower, as presented in Tables 7 and 8.

Next, we compare the performance of the KPRO method with those of the methods introduced in other studies. We consider sentiment classification using the SST-2 dataset. In 2014, Yoon^12^ achieved the best accuracy (88.1%) using a CNN method. In 2019, Jacob et al.^7^ achieved an accuracy of 94.9% using the BERT method. The improvement in accuracy was approximately 6.8%. The accuracies achieved by Lan et al.^9^ (95.2%) and Yang et al.^10^ (96.8%) were 7.1% and 8.7% higher than that achieved by Kim^12^ (88.1%), respectively. McCann et al.^13^ demonstrated the superiority of their model over Kim's CNN^12^ by achieving an accuracy improvement of approximately 3.1% (91.2% versus 88.1%). Benlahbib and Nfaoui^15^ combined some data processing proposals with BERT's WordPiece; they achieved a relatively low accuracy on the IMDb dataset (88.81%), which was lower than that reported by Rehman et al.^1^ (91%). Furthermore, when the highest document-level sentiment-classification accuracy on the raw OD2 dataset was 71.39%, the KPRO method improved it by 18.71% (refer to Table 8) using a basic CNN model. The lowest improvement was 16.68% (88.07% versus 71.39%), which was obtained using the LSTM method or CNN-Bi-LSTM model. Notably, the CNNs delivered an accuracy of 71.39% in the grading test on the OD2 raw dataset, which is also reasonable, compared to those achieved by Yin et al.^20^ and Mukhlash et al.^21^.

The accuracies of the SVM and naïve Bayes algorithms were not as high as those of the deep learning algorithms. However, their accuracy improvements were significant. The SVM and naïve Bayes algorithms improved the accuracy by 13.95% and 11.55%, respectively, when trained using data processed by the KPRO method, in comparison with those obtained when raw data were used for training. The BERT method improved the accuracy by 19.76% when trained on the FCC2 dataset, in comparison with that obtained when trained on the OD2 dataset (82.16% versus 62.40%).

With the sentiment classification experiment for the dataset using three sentiment polarities (Table 7), the best accuracy of the deep learning algorithms in sentiment classification when trained with raw data was 63.22%. The deep learning algorithms improved the accuracy to the highest level of 17.80% (in the case of using the CNN algorithm, it was 81.02%) when trained with data processed by KPRO. The lowest improvement in accuracy was 16.58% (for the CNN-LSTM model, which achieved an accuracy of 79.80%).

## Conclusion and future work

This paper proposes that the KPRO method helps deep learning algorithms to understand the word-level language through a calculation process using the word2vec tool to enhance learning algorithms in terms of both aspect-level and document-level sentiment classifications. The efficiency of comment classification is evident when sentiments are classified at the document level. The time cost of the KPRO method is substantial but only in the data preprocessing stage; thus, it will not be an obstacle for the exploitation of deep learning algorithms. The obtained positive results imply that the KPRO method will help utilize deep learning methods for solving the problem of sentiment classification in new domains, specialized domains, and domains that do not have enough data easily and with higher efficiency. The feature can exploit the knowledge of experts in a specific field, thereby considerably improving the performance of deep learning algorithms with only the basic configuration of the KPRO method. Thus, sentiment analysis can be applied to real-world problems with high confidence for new datasets. We hope that the proposed data processing model can provide a new approach for improving deep learning methods for sentiment analysis.

This study tested the KPRO method with the computational feature technique of BERT, word2vec, and compared it with the tf-idf method. The follow-up studies will aim to evaluate the effectiveness of the proposed method on other word-embedding models and investigate other topics and problems in natural language processing. In addition, we will also study safety issues related to deep learning systems, as noted by Chen et al.^36^, for the proposed KPRO method of this study.

## Data Availability

The codes can be obtained from the corresponding author.
